# γH2AX and Chk1 phosphorylation as predictive pharmacodynamic biomarkers of Chk1 inhibitor-chemotherapy combination treatments

**DOI:** 10.1186/1471-2407-14-483

**Published:** 2014-07-04

**Authors:** Rebecca Rawlinson, Andrew J Massey

**Affiliations:** 1 Vernalis R&D Ltd, Granta Park, Cambridge, UK CB21 6GB

**Keywords:** Chk1, DNA damage, Biomarker, Combination therapy

## Abstract

**Background:**

Chk1 inhibitors are currently in clinical trials in combination with a range of cytotoxic agents and have the potential to potentiate the clinical activity of a large number of standard of care chemotherapeutic agents. Utilizing pharmacodynamic biomarkers to optimize drug dose and scheduling in these trials could greatly enhance the likelihood of clinical success.

**Methods:**

In this study, we evaluated the *in vitro* potentiation of the cytotoxicity of a range of cytotoxic chemotherapeutic drugs by the novel Chk1 inhibitor V158411 in p53 mutant colon cancer cells. Pharmacodynamic biomarkers were evaluated *in vitro*.

**Results:**

V158411 potentiated the cytotoxicity of a range of chemotherapeutic agents with distinct mechanisms of action in p53 mutant colon cancer cell lines grown in anchorage dependent or independent culture conditions. Analysis of pharmacodynamic biomarker changes identified dependencies on the chemotherapeutic agent, the concentration of the chemotherapeutic and the duration of time between combination treatment and biomarker analysis. A reduction in total Chk1 and S296/S317/S345 phosphorylation occurred consistently with all cytotoxics in combination with V158411 but did not predict cell line potentiation. Induction of γH2AX levels was chemotherapeutic dependent and correlated closely with potentiation of gemcitabine and camptothecin in p53 mutant colon cancer cells.

**Conclusions:**

Our results suggest that Chk1 phosphorylation could be a useful biomarker for monitoring inhibition of Chk1 activity in clinical trials involving a range of V158411-chemotherapy combinations and γH2AX induction as a predictor of potentiation in combinations containing gemcitabine or camptothecin.

## Background

The DNA damage response (DDR) is a complex network of signaling pathways that have evolved to protect cells from DNA damage or interference with DNA synthesis. A series of cell cycle checkpoints at G1/S, intra-S or S, and G2/M protect cells from undergoing aberrant division in the presence of DNA damage thereby allowing DNA repair, regulation of transcription and apoptosis [1-4]. The serine-threonine checkpoint kinases Chk1 and Chk2 are often described as the “central transducers” of the DDR and are activated by the ATM kinase in response to DNA breaks and ATR kinase by single-stranded regions of DNA and form the key link between the sensing kinases ATM/ATR and the cell cycle machinery. Recognition of DNA double strand breaks by the Mre11 complex (Mre11, Rad50 and Nbs1) or replication stress by the Rad9-Hus1-Rad1 complex results in the activation of the ATR and ATM kinases respectively. These kinases, in turn, activate the effector kinases Chk1 and Chk2. Chk1 activation occurs predominantly by three phosphorylation events on S317 and 345 by ATR [5,6] and auto-phosphorylation on S296 [7]. Chk1 and Chk2 negatively regulate the Cdc25 family of phosphatases thereby preventing cell cycle progression as well as directly modulating repair proteins resulting in effective lesion repair. Biochemical and genetic studies have demonstrated Chk1 to be essential and indispensable for the S- and G2/M checkpoints [1,8]. In the vast majority of human cancers, p53 (an important effector of the G1/S checkpoint) is mutated or functionally inactivated, rendering cancer cells reliant on Chk1/Chk2 for checkpoint activation, in the presence of endogenous or exogenous DNA damage.

DNA damaging cytotoxic chemotherapeutic agents and ionizing radiation are the mainstay of current cancer treatment regimens. These agents target the DNA in cancer cells and induce DNA damage either directly through DNA adduct formation (for example cisplatin) or indirectly via inhibition of DNA synthesis (for example gemcitabine and 5-fluoruracil) or DNA unwinding (for example etoposide). All of these processes result in DNA strand breaks, activation of the DDR and cell cycle checkpoints, and ultimately cell cycle arrest. Targeting the DDR through Chk1 inhibition, therefore, represents a novel therapeutic strategy to increase DNA-damaging chemotherapeutic drug induced tumor cell death in p53 pathway defective cancers [9,10] by abrogating the remaining intact checkpoint. This “synthetic lethality” approach should increase the therapeutic index of a given chemotherapeutic drug as normal cells remain protected by their functional p53 pathway. This approach has started to be tested clinically with multiple small molecule inhibitors of Chk1 in clinical evaluation in Phase I (GDC-0425 and GDC-0575) or Phase II (LY2603618 [11] and MK-8776 (SCH 900776) [12]) trials in combination with gemcitabine, pemetrexed and cisplatin [13].

The advent of molecularly targeted cancer therapeutics has resulted in increased emphasis on identifying pharmacological biomarkers of drug/target interaction to help accelerate the progress of novel agents through clinical trials [14-16]. To date, biomarker and clinical studies of Chk1 inhibitors have predominantly focused on the combination with gemcitabine. However, Chk1 inhibitors have the potential to be combined with a wide range of cytotoxic chemotherapeutics. In this study, we evaluated the potential for a novel, highly selective Chk1/2 inhibitor, V158411, to potentiate the cytotoxicity of a range of agents in p53 mutant colon cancer cells and the corresponding changes in a panel of potential pharmacodynamic biomarkers for predictors of V158411 combinatorial activity.

## Methods

### Cell lines and cell culture

All cell lines were purchased from the American Type Culture Collection (ATCC), established as a low passage cell bank and then routinely passaged in our laboratory for less than 3 months after resuscitation. HT29, Colo205 and HCT116 cells were routinely cultured in DMEM containing 10% FCS and 1% penicillin/streptomycin at 37°C in a normal humidified atmosphere supplemented with 5% CO_2_.

### Compounds

Solid stocks were purchased from the indicated suppliers and prepared as concentrated stock solutions in the appropriate solvent: gemcitabine (Apin Chemicals Inc), 20 mM in H_2_O; camptothecin (LC Laboratories), 5 mM in DMSO; cisplatin (David Bull Laboratories), 3.33 mM in 1% NaCl in H_2_O; oxaliplatin (Tocris), 5 mM in H_2_O; etoposide (Selleckchem), 20 mM in DMSO; doxorubicin (Selleckchem), 5 mM in DMSO; 5-fluorouracil (Sigma), 50 mM in DMSO; LY2603618 (Selleckchem), 20 mM in DMSO and MK-8776 (ChemieTek), 20 mM in DMSO.

### Potentiation assays

5000 cells per well were seeded in 96-well plates and incubated overnight. Cells were treated with a 10-point titration of cytotoxic chemotherapeutic agent in the presence of a fixed concentration of Chk1 inhibitor for 72 or 168 hours. The effect on cell proliferation was determined using CellTiter 96 AQueous One Solution Cell Proliferation Assay (MTS, Promega) and read on a Victor plate reader (Perkin Elmer).

### Anchorage independent growth assays

1500 cells/well in 0.4% low melting point agarose (SeaPlaque, Lonza) in complete media were plated on to 96-well plates coated with 0.8% low melting point agarose in complete media. Wells were subsequently overlaid with complete media containing cytotoxic chemotherapeutic agents and Chk1 inhibitor. Following incubation for 168 hours, cell viability was determined using CellTiter Blue (Promega) and fluorescence determined using a Victor plate reader (Perkin Elmer).

### Spheroid growth assays

Multi-cellular tumor spheroid assays were preformed essentially as described previously [17]. 1000 HT29 cells/well were seeded in 96-well round bottomed ultra-low attachment microplates (Corning Costar), centrifuged at 1000 × g for 3 minutes and spheroids formed for 72 hours. Spheroid cell viability after incubation with chemotherapeutic drug plus V158411 for 168 hours was determined using CellTiter-Glo Luminescent Cell Viability Assay (Promega).

### Immunoblotting

Antibodies against Chk1, pChk1 (S317), pChk1 (S345), pChk2 (T68), pChk2 (S516), γH2AX, pCdc2 (Y15), pCdc25c (S216), Cdc25a, phH3 (S10), PARP, cleaved PARP, 53BP1, cyclin A, cyclin B1, cyclin D, cyclinE, pCDK2 (T160) and RPA70 were purchased from Cell Signaling Technologies and pChk1 (S296) from Abcam.

Cells were washed once with PBS and lysed in RIPA buffer containing protease and phosphatase inhibitor cocktails (Roche). Protein concentration was determined using a BCA kit (Pierce). Equal amounts of lysate were separated by SDS-PAGE and western blot analysis conducted using the antibodies indicated above

### Statistical analysis

Results were analyzed using a Student’s t-Test tool within the data analysis package provided by Microsoft Excel.

### Ethical approval

None of the research in this manuscript involved human subjects, human material, or human data, or used regulated vertebrates or invertebrates.

## Results

### V158411 potentiates the cytotoxicity of chemotherapeutic agents in p53 mutant colorectal cancer cell lines

**Figure 1:**
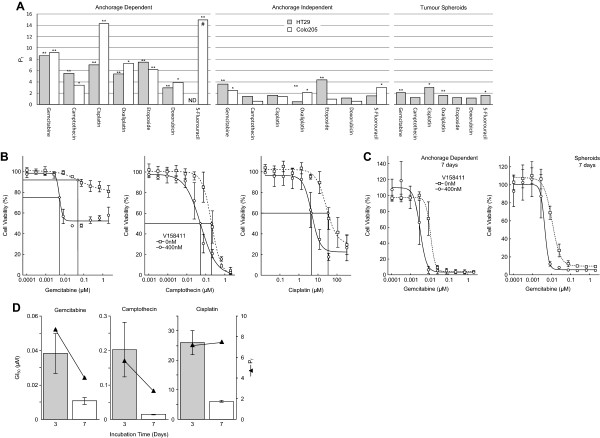
**Determination of *in vitro *potentiation of cytotoxic chemotherapeutic agents by V158411. A**. Comparison of potentiation factors in HT29 and Colo205 cancer cells grown anchorage dependently, anchorage independently or as multicellular tumor spheroids for cytotoxic chemotherapeutic agents with 400 nM V158411. Pf values were calculated from the average GI_50_ and combination GI_50_ (cGI_50_) of at least three determinations where Pf equals average GI_50_/average cGI_50_. *, *P* < 0.05; **, *P* < 0.01; ND, not determinable; #, Pf > 15. **B**. 72 hour HT29 dose response curves for gemcitabine, camptothecin and cisplatin in combination with DMSO or 400 nM V158411 demonstrating the calculation of the GI_50_ (square and dotted line) and cGI_50_ (circle and line). **C**. HT29 dose response curves for gemcitabine following 168 hour treatment with DMSO or 400 nM V158411. HT29 cells were grown either anchorage dependently (left graph) or as multi-cellular tumor spheroids (right graph). **D**. Comparison of the single agent GI_50_ and consequent potentiation factor (Pf, triangle and line) following either 72 hour (3 day) or 168 hour (7 day) incubation with gemcitabine, camptothecin or cisplatin with 0 or 400 nM V158411 in HT29 cells growing anchorage dependently. Values are the average of at least 3 determinations ± SD.

V158411 is a potent, selective inhibitor of recombinant Chk1 and Chk2 kinases *in vitro* with IC_50_s of 3.5 and 2.5 nM [18] respectively but demonstrates a 19-fold cellular selectivity for Chk1 over Chk2. V158411 potentiated the cytotoxicity of a range of chemotherapeutic agents in the p53 mutant colon carcinoma cell lines HT29 and Colo205 growing anchorage dependently, anchorage independently or as multi-cellular tumor spheroids (Figure 1A and 1B). The p53 mutant HT29 colon carcinoma cell line has been extensively used to evaluate the potentiation of cytotoxic chemotherapy by Chk1 inhibitors [19-23] and was therefore used as the main test system for this study. Greater potentiation was observed in both cell lines growing anchorage dependently compared to anchorage independently. The combination treatment of V158411 with gemcitabine not only reduced the concentration of gemcitabine required to inhibit the growth of HT29 or Colo205 cells but also markedly reduced the viability of cells treated with gemcitabine (Figure 1B). This effect was only observed for the shorter 72 hour incubation. Gemcitabine, when incubated for a longer time of 168 hours, induced greater degrees of cell death that was not further potentiated by the addition of V158411 (Figure 1C). The magnitude of potentiation of chemotherapeutic agent cytotoxicity by V158411 was dependent on exposure time for some agents. For example, in HT29 cells growing anchorage dependently, increased exposure time to gemcitabine or camptothecin resulted in increased cytotoxicity of the chemotherapeutic agents as single agents and subsequent reduced potentiation by V158411 (Figure 1D). In contrast, the potentiation of cisplatin cytotoxicity by V158411 remained unchanged following longer incubation.

### DNA damage checkpoint activation is cytotoxic chemotherapeutic agent dependent

**Figure 2:**
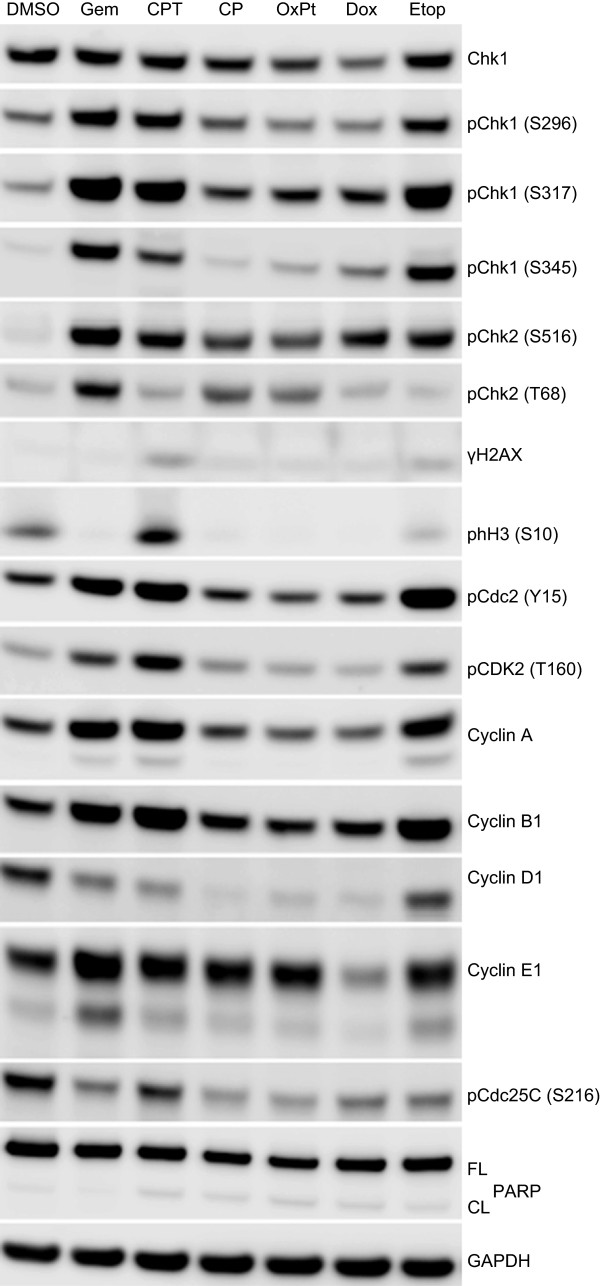
**Checkpoint activation and DNA damage protein biomarker responses in HT29 cells following treatment with cytotoxic chemotherapeutic agents.** HT29 cells were treated with approximately 5-times the single agent GI_50_ of gemcitabine (Gem, 0.2 μM), camptothecin (CPT, 1 μM), cisplatin (CP, 125 μM), oxaliplatin (OxPt, 250 μM), doxorubicin (Dox, 3 μM) or etoposide (Etop, 50 μM) for 24 hours. Changes in protein expression levels were determined by immunoblotting.

The ability of an equitoxic concentration of gemcitabine, camptothecin, cisplatin, oxaliplatin, doxorubicin or etoposide to induce protein biomarker changes associated with activation of the DNA damage checkpoint was evaluated in HT29 cells. Protein biomarker changes induced in HT29 cells were dependent on the chemotherapeutic agent. Gemcitabine, camptothecin and etoposide treatment increased Chk1 phosphorylation at S296, S317 and S345, phosphorylation of Cdc2 at Y15, and CDK2 at T160 and increased total cyclin A and B1 protein levels (Figure 2). In comparison, cisplatin, oxaliplatin and doxorubicin treatment also increased Chk1 S317 phosphorylation but not S296 or S345 phosphorylation. Cyclin D1 levels were also reduced with these three agents whilst cyclin A and B1 levels remained unchanged. These differences in protein biomarker changes may be a reflection of the differential cell cycle perturbations induced by the various chemotherapeutic drugs.

### V158411 inhibits DNA damage induced Chk1 auto-phosphorylation and increases γH2AX in colon carcinoma cells

**Figure 3:**
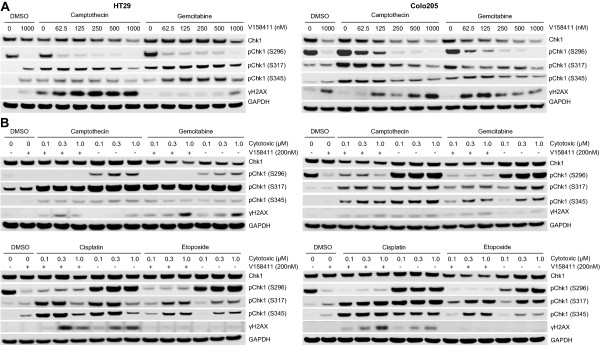
**V158411 inhibits DNA damage induced Chk1 auto-phosphorylation and increases γH2AX in colon carcinoma cells.** HT29 (left) or Colo205 (right) p53 defective colon carcinoma cells were treated with **A**. 200 nM camptothecin or 100 nM gemcitabine plus varying concentrations of V158411 for 24 hours or **B**. treated with the indicated concentrations of gemcitabine, camptothecin, cisplatin or etoposide with either DMSO or 200 nM V158411 for 24 hours. Protein expression was characterized by immunoblotting.

**Figure 4:**
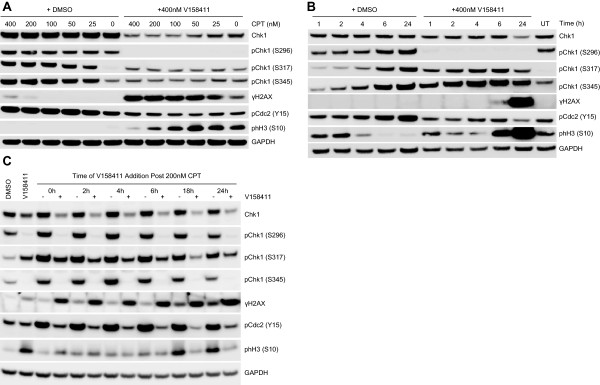
**Pharmacodynamic changes to DNA damage checkpoint and cell cycle proteins in HT29 colon cancer cells by V158411 in combination with camptothecin. A**. HT29 cells were treated with 0 to 400 nM camptothecin (CPT) in the presence of DMSO or 400 nM V158411 for 24 hours. **B**. HT29 cells were treated with 100 nM camptothecin plus DMSO or 400 nM V158411 for 1 to 24 hours. UT, untreated control. **C**. HT29 cells were treated with 200 nM camptothecin for 0 to 24 hours followed by 400 nM V158411 (+) or DMSO (−) for a further 24 hours. Protein expression was characterized by immunoblotting.

To determine the most appropriate biomarkers of V158411 activity, p53 mutant HT29 and Colo205 colon carcinoma cells were treated with a fixed dose of either gemcitabine or camptothecin with an increasing concentration of V158411 for 24 hours (Figure 3A). V158411 induced a dose dependent decrease in Chk1 auto-phosphorylation (at S296) and an increase in γH2AX in HT29 and Colo205 cells in combination with gemcitabine and camptothecin. In HT29 cells, the induction of γH2AX by V158411 was more pronounced in combination with camptothecin than with gemcitabine whilst in Colo205 cells, treatment with V158411 induced γH2AX by roughly equal amounts in combination with either gemcitabine or camptothecin. Exposure of HT29 or Colo205 cells to increasing concentrations of gemcitabine, camptothecin, cisplatin or etoposide in the presence of a fixed concentration of V158411 for 24 hours, resulted in reduced pChk1 (S296) with all four cytotoxic agents (Figure 3B). γH2AX induction was less apparent and appeared dependent on cytotoxic agent and concentration. Chk1 inhibition in combination with chemotherapy induced DNA damage was predicted to have dramatic effects on replication fork stability and cell cycle arrest. However, at the time point studied, no difference in pChk1 (S317) or pChk1 (S345) levels could be perceived between cells treated with DNA damaging agent alone or in combination with V158411. To understand in more detail the biomarker responses therefore following cytotoxic chemotherapy in combination with V158411, we evaluated the effect of concentration, time and schedule on camptothecin induced biomarker responses. Inhibition of Chk1 S296, S317 and S345, and Cdc2 Y15 phosphorylation as well as γH2AX induction by V158411 occurred at a wide range of camptothecin concentrations from 25 to 400 nM (Figure 4A). Treatment with camptothecin followed by V158411 induced an abrogation of DNA damage induced arrest and an increase of cells into mitosis (determined by a decrease in pCdc2 (Y15) and an increase in phH3 (S10)) compared to treatment with camptothecin alone.Duration of exposure to V158411 and camptothecin, but not schedule of administration, had a significant effect on observed biomarker changes. Inhibition of Chk1 S296 phosphorylation occurred within 1 hour of V158411 administration and was maintained for the whole 24 hours. In the presence of camptothecin, V158411 induced rapid phosphorylation (within 1 hour) of Chk1 at S317 and S345 that reached a maximum by 6 hours and then decreased again by 24 hours. By comparison, in the absence of V158411, the induction of phosphorylation of Chk1 at S317 and S345 by camptothecin was significantly delayed. The kinetics of γH2AX and phH3 (S10) induction lagged behind Chk1 S317/S345 phosphorylation being detectable by 6 hours and reaching maximal induction by 24 hours (Figure 4B). Administering V158411 either immediately or up to 24 hours after camptothecin did not result in a different pattern of biomarker changes (Figure 4C). In all combination treatment regimens, total Chk1 as well as phospho-Chk1 S296, S317 and S345 were decreased and γH2AX increased compared to camptothecin treatment alone.

**Figure 5:**
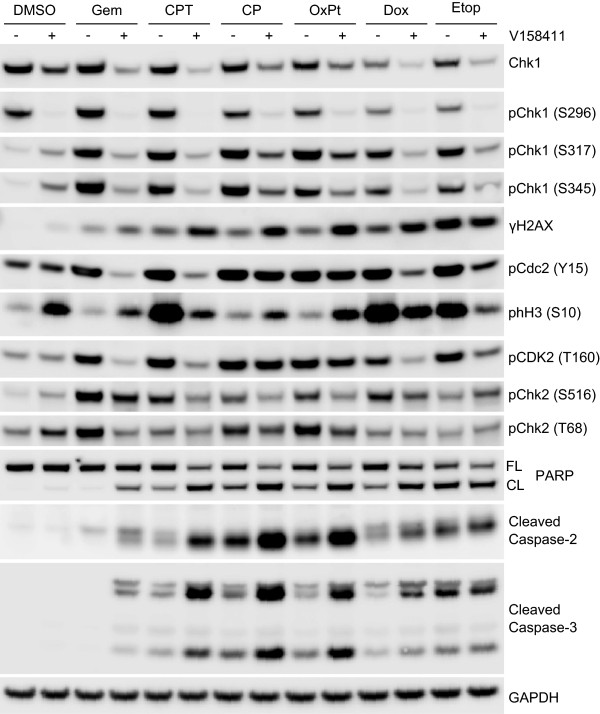
**Cellular biomarker responses in HT29 cells exposed to various cytotoxic chemotherapeutic agents in combination with the Chk1 inhibitor V158411.** HT29 cells were exposed to the combination GI_80_ of gemcitabine (0.2 μM), camptothecin (0.44 μM), cisplatin (68 μM), oxaliplatin (131 μM), doxorubicin (1.2 μM) or etoposide (59 μM) for 18 hours followed by DMSO (−) or 400 nM V158411 (+) for a further 24 hours. Protein expression was characterized by immunoblotting.

To assess the specificity of pChk1 and γH2AX as biomarkers of cytotoxic chemotherapy plus V158411 combination activity, HT29 cells were treated with the combination GI_80_ of gemcitabine, camptothecin, cisplatin, oxaliplatin, doxorubicin or etoposide for 18 hours followed by 0 or 400 nM V158411 for a further 24 hours. In combination with all cytotoxics, V158411 decreased total Chk1 protein levels as well as pChk1 S296, S317 and S345. γH2AX was induced by V158411 in combination with all agents except etoposide which caused a significant increase in γH2AX as a single agent (Figure 5). V158411 reduced Cdc2 Y15 and CDK2 T160 phosphorylation following gemcitabine, camptothecin, doxorubicin and etoposide treatment but not with either of the platinating agents. Changes to histone H3 phosphorylation were chemotherapeutic agent dependent as were the changes to Chk2 phosphorylation. Increased apoptosis, as measured by cleavage of caspase-2 and −3, has been suggested to be a potential biomarker of Chk1 inhibitor induced chemosensitization. Increases in PARP, caspase-2 and caspase-3 cleavage was observed following treatment of HT29 cells with gemcitabine, camptothecin, cisplatin, oxaliplatin and doxorubicin but not etoposide in combination with V158411 compared to cytotoxic treatment alone.

**Figure 6:**
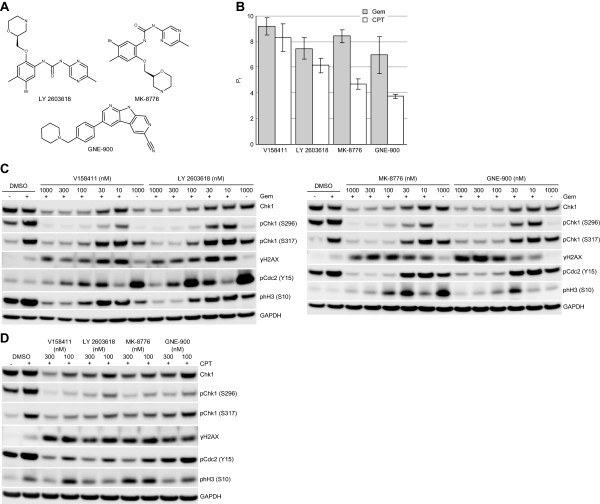
**Potentiation of gemcitabine and camptothecin cytotoxicity and protein biomarker changes induced in HT29 cells by structurally diverse Chk1 inhibitors. A**. Chemical structures of Chk1 inhibitors. **B**. Potentiation of gemcitabine (Gem) or camptothecin (CPT) cytotoxicity in HT29 cells following 72 hour exposure in combination with 300 nM V158411, LY2603618, MK-8776 or GNE-900. The potentiation factor (Pf) was calculated as GI_50_ cytotoxic agent alone/GI_50_ cytotoxic agent plus Chk1 inhibitor. Values represent the average of 3 determinations ± SD. **C**. Biomarker changes induced in response to gemcitabine plus Chk1 inhibitor treatment in HT29 colon carcinoma cells. HT29 colon cancer cells were exposed to 50 nM gemcitabine (+) for 16 hours followed by increasing concentrations of Chk1 inhibitor for a further 24 hours. Protein expression was characterized by immunoblotting. **D**. Biomarker changes induced in response to camptothecin plus Chk1 inhibitor treatment in HT29 colon carcinoma cells. HT29 colon cancer cells were exposed to 100 nM camptothecin for 16 hours followed by 100 or 300 nM Chk1 inhibitor for a further 24 hours.

The specificity of γH2AX and Chk1 phosphorylation as general biomarkers of Chk1 inhibitors was evaluated using three additional, structurally diverse Chk1 inhibitors: LY2603618 [11], MK-8776 (SCH 900776) [12] and GNE-900 [24] (Figure 6A). LY2603618, MK-8776 and GNE-900 potentiated the cytotoxicity of gemcitabine and camptothecin to HT29 colon carcinoma cells comparably to V158411 in terms of concentration and level of potentiation (Figure 6B). Biomarker responses were subsequently evaluated in HT29 cells treated with gemcitabine or camptothecin in combination with V158411, LY2603618, MK-8776 or GNE-900. In combination with gemcitabine or camptothecin, 100 or 300 nM V158411, LY2603618, MK-8776 and GNE-900 induced a dose dependent decrease in Chk1 phosphorylation at S296 and S317 and a concomitant increase in γH2AX protein (Figure 6C and 6D) compared to camptothecin alone.

### Induction of γH2AX by V158411 in combination with cytotoxic chemotherapy in colon carcinoma cells is p53 status dependent

**Figure 7:**
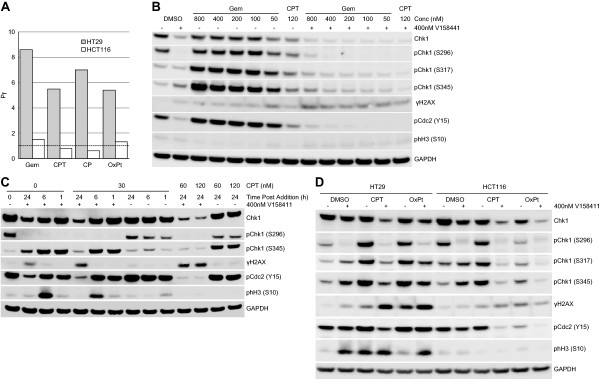
**Comparison of protein biomarker changes in p53 proficient and deficient colon cancer cell lines. A**. Potentiation of the cytotoxicity of gemcitabine (Gem), camptothecin (CPT), cisplatin (CP) or oxaliplatin (OxPt) by 400 nM V158411 was determined in p53 mutant HT29 or p53 wild-type HCT116 colon cancer cells after 72 hours. GI_50_ and cGI_50_ were calculated from the dose response curves using XLFit. The potentiation factor (Pf) was calculated as GI_50_/cGI_50_. Protein biomarker changes were assessed in HCT116 cells treated with **B**. 50 to 800 nM gemcitabine or 120 nM camptothecin in combination with 0 (−) or 400 nM (+) V158411 for 24 hours or **C**. camptothecin plus 0 (−) or 400 nM (+) V158411 for various time combinations and dosing regimens. **D**. HT29 or HCT116 cells were treated with the single agent IC_80_ of camptothecin (HT29, 0.43 μM; HCT116, 0.44 μM) or oxaliplatin (HT29, 131 μM; HCT116, 74 μM) for 18 hours followed by DMSO (−) or 400 nM (+) V158411 for a further 24 hours. Protein expression was characterized by immunoblotting.

Potentiation of gemcitabine, camptothecin, cisplatin and oxaliplatin cytotoxicity by V158411 was dependent on p53 status. In the p53 wild-type colon cancer cell line HCT116, V158411 did not potentiate any of the four cytotoxic chemotherapeutic agents tested (Figure 7A). In comparison, V158411 potentiated all four agents by greater than 5-fold in the HT29 colon carcinoma cell line which harbors a R273H mutation in p53. The pharmacodynamic biomarker response of HCT116 cells to either gemcitabine or camptothecin in combination with V158411 was evaluated. In combination with gemcitabine, V158411 treatment reduced Chk1 auto-phosphorylation in addition to total Chk1 protein levels and decreased phosphorylation of Cdc2 at Y15. These changes to Chk1 activity however, did not result in an increase in γH2AX (Figure 7B). Likewise, in combination with camptothecin, V158411 treatment again resulted in a decrease in pChk1 (S296), total Chk1 and pCdc2 (Y15) and no change in γH2AX protein levels compared to DMSO or V158411 treatment alone (Figure 7C). The response of a wide range of biomarkers in HT29 and HCT116 cells to camptothecin or oxaliplatin in combination with V158411 was evaluated. As was observed previously, in the p53 mutant HT29 cells, V158411 in combination with either camptothecin or oxaliplatin reduced Chk1 phosphorylation at S296, S317 and S345 as well as total Chk1 protein levels resulting in increased γH2AX. In HCT116 cells, similar changes to Chk1 protein levels and phosphorylation were observed but in this cell line did not correlate with an increase in γH2AX (Figure 7D). Whilst the HT29 and HCT116 cell lines are both derived from the same tissue type, they are non-isogenic and further studies are warranted in matched isogenic cell line pairs.

### Potentiation of gemcitabine and camptothecin cytotoxicity by V158411 occurs independently of fetal calf serum or oxygen concentration and under anchorage independent growth conditions

**Figure 8:**
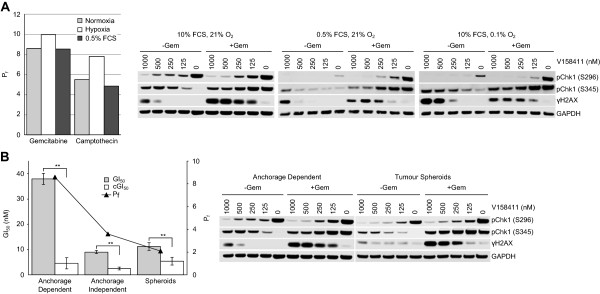
**Potentiation of gemcitabine and camptothecin cytotoxicity by V158411 occurs independently of fetal calf serum or oxygen concentration and under anchorage independent growth conditions. A**. Potentiation of the cytotoxicity of gemcitabine or camptothecin by 400 nM V158411 was determined in HT29 cells growing in 10% FCS, 21% O_2_ (normoxia); 10% FCS, 0.5% O_2_ (hypoxia) or 0.5% FCS, 21% O_2_ for 72 hours. GI_50_ and cGI_50_ were calculated from the dose response curves using XLFit. The potentiation factor (Pf) was calculated as the average GI_50_/average cGI_50_ from 3 determinations. Protein biomarker changes were subsequently assessed in HT29 cells growing under the same conditions following treatment with 100 nM gemcitabine plus 0 to 1000 nM V158411 for 24 hours. **B**. HT29 cells growing either attached to plastic cell culture plates (anchorage dependently), anchorage independently in LMP agarose or as multicellular tumor spheroids were exposed to increasing concentrations of gemcitabine in the presence or absence of 400 nM V158411 for 72 (anchorage dependent) or 168 (anchorage independent/spheroid) hours. **, *P* < 0.01. Protein biomarker changes were assessed in HT29 cells growing anchorage dependently or as multi-cellular tumor spheroids following treatment with 100 nM gemcitabine plus 0 to 1000 nM V158411 for 24 hours. Protein expression was characterized by immunoblotting.

The ability of V158411 to potentiate gemcitabine or camptothecin cytotoxicity under conditions that more closely reflect *in vivo* tumor growth conditions, namely anchorage-independent growth, hypoxia or low nutrient growth conditions, were evaluated. Potentiation of gemcitabine or camptothecin cytotoxicity by V158411 was unaffected by growth of HT29 cells under low (0.5%) FCS or hypoxic (0.1% O_2_) conditions (Figure 8A). Under hypoxic growth conditions, the potentiation of gemcitabine or camptothecin cytotoxicity increased from 8.6 to 10.0-fold for gemcitabine and from 5.5 to 7.8-fold for camptothecin compared to normoxic growth. Likewise, in low FCS growth conditions, the potentiation of gemcitabine or camptothecin cytotoxicity by V158411 remained approximately equal to that induced in high FCS growth conditions (8.5 versus 8.6-fold for gemcitabine and 4.9 versus 5.5-fold for camptothecin). In combination with gemcitabine, V158411 reduced pChk1 (S296) and increased pChk1 (S345) and γH2AX protein levels in HT29 cells grown under normoxic, hypoxic or low FCS conditions (Figure 8A).V158411 potentiated the cytotoxicity of gemcitabine in HT29 cells growing anchorage dependently or independently (Figure 8B). The potentiation observed in HT29 cells in anchorage dependent culture (Pf 8.6) was greater than that observed in anchorage independent growth in low melting point agarose (Pf 3.6) or as multi-cellular tumor spheroids (Pf 2.1). In HT29 multi-cellular tumor spheroids, the combination treatment of gemcitabine plus V158411 reduced Chk1 auto-phosphorylation (S296) and increased Chk1 phosphorylation at S345 and γH2AX levels albeit at higher concentrations of V158411 than that required under normal anchorage dependent growth conditions (Figure 8B).

## Discussion

Seven structurally distinct inhibitors of the serine/threonine checkpoint kinase Chk1 have been evaluated or are currently being actively tested in combination clinical trials with a range of cytotoxic chemotherapy drugs such as irinotecan, cisplatin, gemcitabine, pemetrexed and cytarabine. These include XL844, AZD7762 and PF477736 which completed Phase I trials and LY2603618 which completed Phase II, but further development of these agents has subsequently been discontinued. GDC-0425 and GDC-0575 continue to be actively developed in a Phase I setting and MK-8776 (SCH 900776) in Phase II. It is interesting to note that all Chk1 inhibitors so far tested in combination clinical trials (seven to date) have undergone clinical testing in combination with gemcitabine (ClinicalTrials.gov) whilst pemetrexed, cisplatin, irinotecan or cytarabine have been tested with only one Chk1 inhibitor each [25-28].

In this study, we evaluated the ability of the novel Chk1 inhibitor V158411 to potentiate the *in vitro* cytotoxicity of seven clinically used cytotoxic chemotherapy drugs with different mechanisms-of-action in two p53 mutant and one p53 wild-type colorectal carcinoma cell lines growing either anchorage dependently, anchorage independently or as multi-cellular tumor spheroids. Analysis of *in vitro* protein biomarker responses was subsequently undertaken in an attempt to identify biomarkers potentially predictive of combinatorial activity.

V158411 induced moderate to good potentiation of all seven cytotoxic agents tested in the two p53 mutant cell lines but not the p53 wild-type cell line. The only exception was with 5-fluorouracil in the HT29 cell line. The lack of potentiation of 5-fluorouracil activity in this cell line was most likely due to the high intrinsic resistance of HT29 cells to 5-fluorouracil (GI_50_ > 100 μM for single agent). Greater potentiation was observed for cells growing anchorage dependently than cells growing either anchorage independently or as multi-cellular tumor spheroids and may be a reflection of the increased rate of proliferation and/or the fraction of cells undergoing active DNA replication in the anchorage dependent culture conditions. In short term combination studies (3 day co-incubation), gemcitabine was the only agent for which V158411 not only potentiated the anti-proliferative activity of the cytotoxic agent but also increased the fraction of cells killed at the higher concentrations of drug. This increased cell killing was only observed for the short 3 day incubation and was subsequently lost at longer incubations possibly due to the increased cytotoxicity of gemcitabine as a single agent.

Previously published studies have observed the greatest potentiation of cytotoxicity by Chk1 inhibitors with the anti-metabolite class of drugs, including gemcitabine [23]. Chk1 activity has been demonstrated to be critical for not only the DNA damage response checkpoint but also for replication fork stabilization, replication origin firing and homologous recombination. These later roles have been suggested to be critical for the increased effectiveness of Chk1 inhibitors in combination with gemcitabine [29] compared to other cytotoxic chemotherapy drugs such as Topoisomerase inhibitors. Gemcitabine inhibits DNA synthesis, DNA replication and cell proliferation through two distinct but linked mechanisms. Gemcitabine diphosphate binds to and irreversibly inhibits ribonucleotide reductase thereby depleting the pool of deoxyribonucleotides available for *de novo* DNA synthesis. The triphosphate analogue of gemcitabine can also be incorporated into DNA (in substitution for cytidine) where it acts as a chain terminator thereby inhibiting further DNA synthesis. Inhibition of ribonucleotide reductase by gemcitabine (or similarly by hydroxyurea) induces replication fork stalling. Chk1 activity is required to maintain replication fork stability and inhibition of Chk1 leads to replication fork collapse and the generation of “new” DNA strand breaks. In p53 mutant cancer cells, the checkpoint is functionally inactivated by Chk1 inhibition, therefore these cells progress through S-phase and enter into a premature, lethal mitosis [30]. Replication fork collapse and checkpoint abrogation by Chk1 inhibitors induces potentially lethal DNA damage killing gemcitabine treated p53-mutant cancer cells by a “double hit” mechanism. It should however be noted that the potentiation of gemcitabine observed in pre-clinical xenograft studies is not nearly as dramatic as that observed in the *in vitro* potentiation studies. The pre-clinical combination studies of gemcitabine in combination with Chk1 inhibitors are generally conducted at gemcitabine concentrations below the gemcitabine maximum tolerated dose and using a schedule (once every 3 days) that is not reflective of the clinical schedule (once weekly). Our studies suggest that other cytotoxic drugs such as cisplatin or oxaliplatin, in addition to gemcitabine, are worthy of further evaluation.

One of the challenges, and goals, of molecularly targeted cancer therapeutic development is the identification of biomarkers (whether they be genetic, protein or macromolecule based) that allow the translation of the understanding and knowledge gained at the molecular and cellular level into a therapy effective for patients [14-16]. These biomarkers can be identified and developed for one of three specific aims: 1, to stratify a patient population into potential responders and non-responders, 2, to ensure adequate target engagement or inhibition at a given dose or 3, to assess for pathway modulation and a potentially positive or beneficial therapeutic outcome. The overall aim of biomarker development and utilization is to accelerate the clinical development and adoption of new anti-cancer therapies.

Previous studies have demonstrated that a deficiency in p53 improves chemopotentiation by Chk1 inhibitors [23]. However, mutation of p53 has been found to be important for overall response but is not sufficient to predict a synergistic outcome between a Chk1 inhibitor and cytotoxic chemotherapy. BRCA, XRCC3, DNA-PK [31] and CYCLIN B1 [32] levels have all been postulated to be important in modulating the effectiveness of a Chk1 inhibitor in combination with a DNA damaging agent. In our study, we attempted to correlate potentiation of DNA damaging agent cytotoxicity by the Chk1 inhibitor V158411 with protein biomarker changes induced by the combination to identify biomarker changes predictive of a robust, combinatorial effect.

Phosphorylation of Chk1 on serine 345, an activation phosphorylation site on Chk1 phosphorylated in response to DNA damage by ATR, correlated closely with response to the combination of gemcitabine plus AZD7762 in pancreatic tumor xenografts [33]. In a separate study, cleaved (activated) caspase-2 levels increased in response to DNA damage when Chk1 was inhibited due to checkpoint inactivation and forced mitotic entry [34]. However, other studies have demonstrated that death induced by the combination of a DNA damaging drug and Chk1 inhibitor was not always dependent on caspase-2 or the PIDDosome [35]. In our study, moderate to high potentiation was observed with all of the DNA damaging agents tested in the p53 mutant but not the p53 wild-type colon cancer cell lines. No unifying biomarker was identified that would appear predictive of effective combinatorial activity. Instead, as might be predicted, biomarker responses appeared DNA damaging agent specific. Additionally, there was dependence on post-treatment time to observe optimal biomarker changes but these responses were less dependent on the schedule of addition of DNA damaging agent and V158411.

In all combinations, V158411 efficiently reduced the levels of auto-phosphorylated (pSer296) Chk1 suggesting that Chk1 was effectively inhibited by the concentration of V158411 utilized. pChk1 (S296) would therefore make a powerful biomarker for ensuring effective target engagement and Chk1 inhibition in clinical samples. In combination with all DNA damaging drugs, V158411 induced a time dependent degradation of Chk1. This may, in part, reflect the normal homeostatic process of cellular checkpoint resetting. A reduction in total Chk1 S317 and S345 phosphorylation occurred most consistently with all cytotoxics in combination with V158411 but did not predict cell line sensitivity as similar biomarker changes were observed in the non-responsive, p53 wild-type HCT116 cell line. Induction of γH2AX expression was chemotherapeutic dependent and correlated closely with potentiation for gemcitabine and camptothecin in p53 mutant but not wild-type colon cancer cells. These protein biomarker changes appeared to not depend on the chemical structure of the CHk1 inhibitor as a similar pattern of changes was observed with a range of Chk1 inhibitors with diverse chemotypes. Assays to measure γH2AX are reasonably well developed and are currently being tested clinically with different cancer therapeutics and may therefor prove a relatively straightforward marker to include in clinical studies [36-38].

## Conclusions

Our results suggest that reduction in Chk1 phosphorylation at serine 296 could be a useful biomarker for monitoring Chk1 activity, and its subsequent inhibition, in clinical trials involving a range of Chk1 inhibitor-chemotherapy combinations. γH2AX induction in combinations containing gemcitabine or camptothecin could potentially serve as a predictive marker of pathway modulation and therapeutic outcome.

## Competing interests

All authors are either present or past employees of Vernalis (R&D) Ltd. and this work was undertaken as part of their employment. AJM is a stock option holder of Vernalis (R&D) Ltd. The authors declare no other competing financial interests. The authors will adhere to the policies outlined on sharing materials, methods and data.

## Authors’ contributions

RR and AJM designed the studies. RR performed the majority of the experiments with help from AJM. RR and AJM wrote the manuscript. All authors read and approved the final manuscript.

## Pre-publication history

The pre-publication history for this paper can be accessed here:

http://www.biomedcentral.com/1471-2407/14/483/prepub
